# Yoga for Health-Related Quality of Life in Adult Cancer: A Randomized Controlled Feasibility Study

**DOI:** 10.1155/2015/816820

**Published:** 2015-06-11

**Authors:** Marcy McCall, Melanie McDonald, Sally Thorne, Alison Ward, Carl Heneghan

**Affiliations:** ^1^Department of Continuing Education, University of Oxford, Oxford OX1 2JA, UK; ^2^Patient and Family Counselling, British Columbia Cancer Agency, Vancouver, BC, Canada V57 1L3; ^3^School of Nursing, University of British Columbia, Vancouver, BC, Canada V6T 2B5; ^4^Department of Primary Care Health Sciences, University of Oxford, Oxford OX2 6NW, UK

## Abstract

An increase in patient-led uptake of complementary therapies in adult cancer has led to a need for more rigorous study of such interventions and their outcomes. This study therefore aimed to evaluate the feasibility and acceptability of a yoga intervention in men and women receiving conventional treatment for a cancer diagnosis. Prospective, mixed methods feasibility trial allocated participants to receive one of three yoga interventions over a four-week study period. Data collection was completed through online survey of QOL-CA/CS and customized surveys. Fifteen participants were included (11 female) undergoing treatment for breast, prostate, colorectal, brain, and blood and lung cancer. Two participants dropped out and complete qualitative and quantitative data sets were collected from 12 participants and four yoga instructors. Other outcome measures included implementation costs patient-reported preferences for yoga intervention and changes in QOL-CA/CS. Three types of yoga intervention were safely administered in adult cancer. Mixed methods, cost-efficiency, QOL-CA/CS, and evidence-based design of yoga intervention have been used to establish feasibility and patient-preferences for yoga delivery in adult caner. Results suggest that, with some methodological improvements, a large-scale randomized controlled trial is warranted to test the efficacy of yoga for male and female cancer patients. This trial is registered with Clinicaltrials.gov NCT02309112.

## 1. Introduction

In 2012, there were 14.1 million new cancer cases and 32.6 million adults living with cancer worldwide [1]. More than 40% of adult cancer patients report frequent usage of complementary and alternative medicine (CAM) to improve symptom management or their health-related quality of life (HRQoL) [2–6]. Complementary therapies (as well as alternative or traditional medicines) are referred to as “a broad set of health care practices that are not part of that country's own tradition and are not integrated into the dominant health care system” [7]. In the United States, yoga is one of the most commonly reported complementary health activities undertaken by individuals [8].

An increase in patient-led uptake of complementary care in adult cancer has led to a need for more rigorous study of such interventions and their outcome effects [9–11]. In terms of yoga research, the volume of literature corresponding to HRQoL and cancer-related symptoms has increased [12, 13]. However, little is known about how the characteristics of yoga and its contextual factors might influence intervention in a clinical setting for adult cancer patients [14]. The few clinical trials of yoga conducted in cancer patients lacked transparency of intervention and adequate randomization [12, 14]. Furthermore, generalizability of study results has been limited due to small samples and because most studies have involved women with breast cancer [15–19].

The purpose of this feasibility study, therefore, was to build knowledge toward designing a randomized controlled trial (RCT) of a yoga intervention to improve HRQoL in adult cancer. This mixed method evaluation of yoga intervention adds perspectives about yoga collected from men and women in receiving conventional treatment for various cancer diagnoses. The study results provide insight into feasibility of yoga intervention in terms of delivery, associated financial expenses, patient adherence, and preferences, as well as exploratory effects on HRQoL in a clinical setting.

## 2. Materials and Methods

The study was designed as a three-arm yoga feasibility trial in adult cancer. This single-centre intervention was conducted in cancer patients (19 years of age or older) receiving or planning to receive conventional treatment that included chemotherapy, radiotherapy, hormone therapy, or active surveillance within 28 days of study enrolment. To participate, patients were first-time or irregular users of yoga (less than two sessions per month over past 12 months) and free of any physical limitation or psychological disturbances that might have interfered with their ability to adhere to protocol or participate in light to moderate physical activity. Patients with pre- or postsurgical intervention were not recruited for this study due to potential complications or concerns about wound healing. Participants agreed to complete online surveys in English and attend in-person meetings in Vancouver, Canada.

All participants were recruited from the Vancouver Centre of the British Columbia Cancer Agency (BCCA) from 15 April 2014 to 30 June 2014 using posted advertisements and referral from health care professionals (HCPs). Interested volunteers contacted the first author (Marcy McCall) through telephone, web-based registration, or email communication. Participants completed a self-report questionnaire to determine their eligibility. Eligible participants advanced to complete online registration and informed consent before documenting personal data and baseline measures of HRQoL.

Participants were randomized into three yoga interventions. A research assistant who was not associated with any other aspect of the study conducted the randomization procedure by physical shuffling of concealed envelopes. The envelopes were color-coded to identify gender and thus stratified accordingly to ensure equal distribution of male and female participants across groups.

Table 1 shows a PaT plot [20] describing the variation of yoga exposure across the three yoga intervention groups. Three evidence-based yoga interventions were designed to vary the amount of exposure, type and number of in-person instructions (see Table 2). The characteristics of yoga intervention were extracted from a literature review that included a component analysis of existing studies that showed positive results on patient-reported outcomes (30 minutes to 120 minutes) [21–24]. A group of certified yoga instructors (*n* = 6) reviewed the framework and finalized the components of the interventions.

The active yoga intervention was implemented for 28 days (four weeks). Feasibility outcome measures were collected using quantitative and qualitative data collection via web-based surveys completed by study participants (12-item Y-ACT survey located at https://www.research.net/s/Y-ACT_SURVEY), yoga instructors (9-item survey located at https://www.research.net/s/INSTRUCTORS_Y-ACT), and email correspondence with clinical administrators. Patients' HRQoL was measured using the QOL-CA/CS [25] instrument (41-item) that was replicated with authorization and adopted to suit an online platform (located at https://www.research.net/s/Y-ACT_QOL-CA-CS).

### 2.1. Ethics Statement

The study was conducted in accordance with a protocol approved by the BCCA Research Ethics Board (5 April 2014, UBC BCCA REB: reference H14-00734) and the Oxford Tropical Research Ethics Committee (OXTREC: reference 534-14). Written informed consent was obtained from patients and personal details of study participants remain confidential. This trial was registered (ClinicalTrials.gov: number NCT02309112) after the data collection was complete for administrative reasons. The authors confirm that all online and related trials for this intervention have been registered.

### 2.2. Data Analysis

Qualitative data were collected through online questionnaires administered to all participants of the study, including patients (*n* = 15) and the yoga instructors (*n* = 4). The three directive open-ended questions asked respondents to comment on their preferences or dislikes about yoga in adult cancer, their suggested improvements to optimize the design of the yoga intervention, and their suggested improvements for aspects of the study design (i.e., recruitment methods and communication material). There was one open-ended item inviting participants to comment on any other aspect of the study that they felt important to share. Individual responses were blinded and analyzed for themes and patterns using an inductive reasoning approach.

Quantitative data from participants were also collected through online questionnaires. The individual data from participants were considered in absolute values. Averages and percentages have been used to describe group data where applicable. Financial costs of the intervention were reported in American currency (USD). An assessment of cost-efficiency across the three interventions divided the total cost of the yoga delivery (including operational expenses such as teaching fees and materials) by the total number of hours of yoga that were delivered to patients in each group (number of hours per participant). The physical space was provided free of charge by the health care facility and not considered in the expenses. Neither researchers' salaries nor volunteers' time have been included in the financial analysis.

## 3. Results

**Figure 1:**
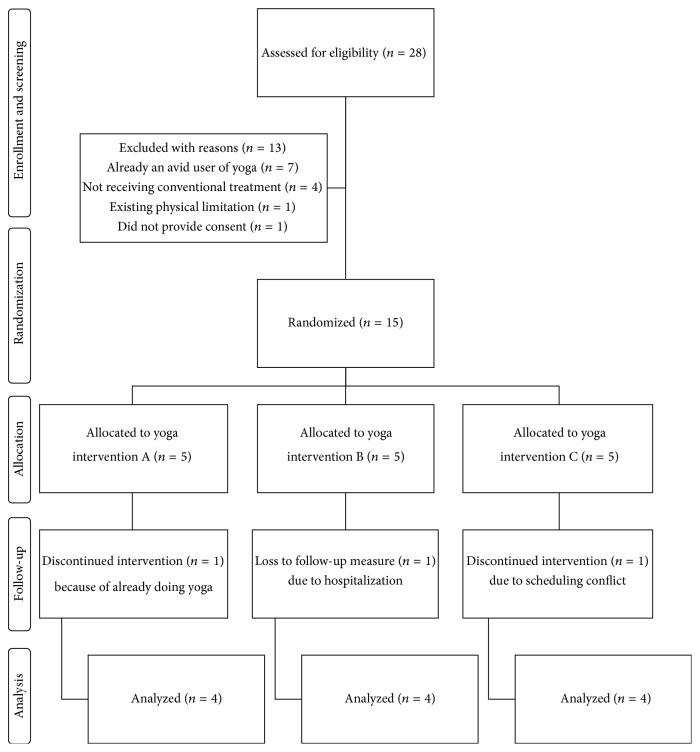
Participant flow diagram.

During the recruitment period (10 weeks), patients contacted researchers through email (*n* = 12), in-person (*n* = 2), a customized webpage (*n* = 12), and telephone (*n* = 4). Of the recruited participants (*n* = 30), 28 patients agreed to enroll in the study and complete the eligibility-screening questionnaire.  Figure 1 details the flow of enrolled participants. Fifteen participants, male (*n* = 4) and female (*n* = 11) patients aged from 33 to 72 years, gave consent to participate in the study.  Table 3 lists characteristics of participants allocated to yoga Groups A (low-dose), B (medium-dose), and C (high-dose). Two participants dropped out of the study after the point of randomization because of scheduling conflict (*n* = 1) and already practising regular yoga (*n* = 1). Thirteen participants (3 male and 10 female) completed the yoga intervention. One additional female participant dropped out of the study due to a cancer-related surgical procedure and did not complete outcome measures. Twelve participants completed the study.

Participants self-reported their diagnoses of cancer, which included breast (*n* = 6), colorectal (*n* = 2), and prostate (*n* = 2) cancer and one of each of lung, skin, tongue, brain, and blood cancer. Thirteen additional male (*n* = 2) and female (*n* = 11) patients volunteered for the study but were ineligible for participation due to their current use of yoga (*n* = 6), having completed conventional treatment (*n* = 4), or not providing consent (*n* = 1).

Table 4 summarizes the participant-reported amount of yoga attendance and satisfaction and its utility across groups. The yoga classes most frequently attended were scheduled on Monday afternoon (20 of 26 possible participants; 77% attendance rate) from 2 p.m. to 3 p.m. and Wednesday evening (16/20; 80%) from 6:30 p.m. to 7:30 p.m. Classes scheduled on Friday at 10 a.m. or 12 p.m. were not as well attended (9/20; 40%).

Across groups, the amount of exposure to yoga ranged from one session (50-minutes) to 24 sessions in four weeks. Table 4 also shows the average satisfaction with the amount of yoga (range 6.8 to 8.5 across groups). Overall, nine participants felt that they were not offered enough yoga; one participant (C3) felt that she was offered “the right amount of yoga” and another (C1) felt that he was offered “too much yoga.” The average satisfaction scores for practising yoga ranged from 6.5 (Group A) to 9 (Group B) and the amount of satisfaction using online yoga varied form 1.7 in Group B to 7.3 in Group A (see Table 4). Interestingly, Group A participants were not provided free access to online yoga following a shorter introductory session (45-minutes).

The average cost of yoga intervention per participant across groups was $360 USD (Group A $255, Group B $325, and Group C $500). This cost included all teaching fees, equipment, and materials to support home-based practice. The most cost-efficient delivery mechanism for in-person yoga was in Group B (100%), where all participants attended the introductory session and workshop (3.5 hours offered). Group C intervention was the least efficient model of delivery where 53% of the available yoga was received by patients (12.5 hours offered). The prepaid online yoga memberships in Groups B and C were not activated by participants and did not appear to increase reports of adherence to home-based yoga practice.

The qualitative analysis of yoga intervention and its appropriateness for this population identified four categories of emergent themes: positive patient experiences, class preferences and characteristics, barriers and their hindrance in yoga participation, and recommendations for future study design.

All responding participants (*n* = 12) appreciated being involved in the study or felt that participating in yoga had been a positive experience. One female participant (A5) said “this study gave me the opportunity to realize that practising yoga can help me in my day to day, feeling more energized and in good spirits.” Respondents described yoga as highly beneficial to their self-care and requested it be offered regularly in their cancer centre. In terms of online yoga, participants in Groups B and C said they were too busy or forgot, and many said they would attempt to activate their online membership now that the study has finished. Participants in Group A identified online yoga as a promising delivery mechanism, helpful in its ability to reduce fear of contracting infection in public space and convenient for hectic treatment schedules.

Patients across groups reported strong preferences for the following components of yoga: small class sizes, cancer-specific group, stretching, breathing practices, meditation, and physical postures for strength conditioning and restorative poses. Several respondents also commented on the quality of yoga instruction and felt the atmosphere was inclusive and agreeable for relaxation. The yoga instructors felt that Group A intervention was too short in duration, Group B's workshop was highly valued, and regular sessions as in Group C were perceived as important to develop content and education and improve participant adherence. The social component of all interventions was reportedly useful and an important opportunity to allow participants to talk about their personal concerns and ask questions.

Fatigue, side effects from conventional treatment, lack of motivation, financial cost, and transportation difficulties were listed barriers for participation by respondents in Groups A and B. Group C respondents generally felt they had competing priorities and felt their work, travel, or scheduling conflicts prevented attendance to yoga in some instances. Online yoga was perceived as “difficult to incorporate” because of lack of space (B3) and lack of time (A1 and B2), or one did not understand instructions to activate the online account (C4).

Several respondents (*n* = 10) offered specific ideas to improve awareness and recruitment of patients to a yoga study. Their ideas included increased promotion and discussion with nurses, oncologists, and general practitioners (GPs), as well as increased word of mouth and advertising via patient support groups and online forums. In terms of yoga delivery, some patients (*n* = 3) felt that yoga intervention should be offered before conventional treatment or surgical operations. One respondent (A5) felt that scheduling regular group meetings would encourage participation and requested more explicit guidance to enhance home-based practice of video or online yoga.

### 3.1. Health-Related Outcomes

No adverse events associated with yoga were reported during the conduct of the study. Two patients (A2 and C2) became hospitalized for causes related to conventional treatment and cancer, including surgery. Twelve participants completed the baseline and outcome measures of QOL-CA/CS. See Table 5 for a summary of results across groups. The average QOL-CA/CS increased from 5.5 to 5.7/10 (0.2 points). The results across the QOL-CA/CS subgroups are presented in Table 6.

## 4. Discussion

All participants who attended an introductory yoga session completed some or all of their additional yoga sessions. Participants reported that the scheduling and location of yoga sessions were “convenient.” Members in all groups expressed likability for yoga intervention, and perceived yoga practice as helpful for relaxation and it provided an opportunity for positive social interaction with other cancer patients.

The dropout rate from previous yoga trials in adult cancer has ranged from 0 to 38% [26]. The dropout rate after the point of randomization in this study was 13% (2 of 15 participants) with one loss to follow-up (7%). Across three yoga groups, the average attendance rate for instructor-led yoga in this study was 80%. This is similar to a pilot of breast cancer survivors (*n* = 12), where the rate of class attendance was 78% (19/24 classes) over a 12-week period [27]. However, as less is known about the long-term yoga attendance rate (>3- to 6-month follow-up), it should be investigated in future studies.

This feasibility trial established that participant screening, registration, and outcome measures for this kind of study can be completed using customized online surveys. The QOL-CA/CS scale has been adapted to an online format and participants who completed the questionnaire in this format did not report dissatisfaction or confusion by its presentation.

With respect to cost, delivery of yoga over a four-week period ranged between $255 USD and $500 USD per participant. This initial cost included teaching fees for the development of class content, staff orientation, yoga instruction, equipment, and material. The intervention expenses could be reduced over time with an increased efficiency in class design and delivery, lower fees for teacher administration, and economies of scales improved with a larger number of participants and amortization of equipment costs. In addition to this, existing nonprofit organizations can deliver yoga to cancer patients at a reduced cost because yoga instructors volunteer their time or offer discounted classes [28].

Results of this feasibility study suggest that free access to online yoga may not increase home-based practice when delivered alongside in-person instruction. A few participants with concerns about scheduling or those with fears to attend public classes due to safety or infection risks said they would be more inclined to adopt online yoga. The potential role of online yoga to alleviate stress in cancer patients (*n* = 47) has been explored elsewhere [29], where results of their protocol development suggested that online classes can increase access to yoga in underserviced geographical regions. Further exploration as to whether unpaid yoga or prepaid online yoga instruction increases adherence to home-based practice for adult cancer patients appears warranted.

In summary, participants and yoga instructors in this study suggested that the following characteristics of yoga intervention were appropriate for adult cancer patients: group classes with in-person instruction (one to two times per week of at least 60-minute duration) and including class components such as breathing techniques, meditation, and physical postures for strength and relaxation. The social interaction in a cancer-specific group has also been identified as a preferred component of yoga intervention. Participants felt the cancer-specific yoga groups alleviated feelings of stress and enhanced motivation to attend yoga regularly. The classes delivered in the earlier part of the week (Monday to Wednesday) initiated between 10 a.m. and 6:30 p.m. had higher attendance rate than classes on Thursday or Friday.

### 4.1. Study Limitations and Recommendations

The exposure and type of yoga was randomly allocated to participants and the outcome investigator of the collected data (Marcy McCall) performed analysis under supervision (Carl Heneghan and Alison Ward). The strength of this study was its application of mixed methods to evaluate characteristics and aspects of yoga intervention that might optimize HRQoL in adult cancer in future research. The small recruitment size (*n* = 30) was an imposed limitation due to the space limitations and budgetary constraints. As a feasibility study, the research objectives did not require a larger sample size, and, therefore, statistical inferences for changes of quality of life should not be implied from this research. Instead, future large-scale designs will consider increasing the duration of the study, with an adequately powered sample size and greater population diversity, perhaps in the context of a single or multicentre clinical trial. Future studies should also seek including post- or presurgical patients, under the medical advice of oncologists.

This current study did not blind patients or the investigators for pragmatic reasons. The recruitment method relied upon patients to self-select, and all types of the data were self-reported. These conditions threaten biased results in the study. In particular, participants who volunteered for the study may have a nonstandard, positive view of yoga; and, second, participants' responses reflect a subjective perception about their experience and their health in one moment in time. Existing methodological weaknesses, therefore, could also be improved by the following design amendments: blind the outcome assessor; include a waitlist or control group with no yoga as in previous studies [30]; provide PaT plot of yoga intervention including 60- to 120-minute sessions one to two times per week over 12-weeks; measure short-term and long-term changes in HRQoL and yoga adherence; consider additional outcome measures such as biomarkers (e.g., cortisol) and changes in health behaviour or treatment response (e.g., patient tolerance of conventional treatment). Additional studies might recruit patients through targeted medical meetings, instead of general postings. The medical meeting recruitment strategy would ensure that patients with health-related limitations or pending surgical interventions do not threaten their ability to adhere to a yoga protocol.

## 5. Conclusion

The results of this feasibility study suggest that adult male and female patients can participate in yoga intervention to improve symptoms related to cancer and its conventional treatment. As in this example, an evidence-based yoga intervention design can improve the appropriateness of and patient adherence to yoga in a clinical setting. A cost analysis and efficiency of three types of yoga intervention have been explored for the first time. The development and implementation of a full-scale randomized controlled trial to explore the efficacy of yoga to improve HRQoL in adult cancer alongside conventional treatment is an appropriate direction for future research.

## Figures and Tables

**Table 1:** PaT plot of yoga intervention.

Timeline	Yoga intervention A	Yoga intervention B	Yoga intervention C
Randomisation			

Baseline	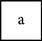	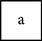	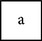

Week 1	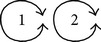	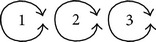	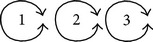

Weeks 2 to 4		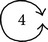	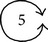

Week 5			

End of study	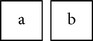	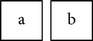	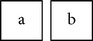

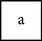	QOL-CA questionnaire completed by consenting participants (online or paper)		

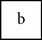	Semi-structured, self-reported survey (Y-ACT) to elicit information regarding at-home yoga adherence, patient experiences, utility/dysutility of yoga, patient attitude and barriers to practising yoga (online or paper)		

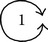	30-minute training session of pranayama (breathing techniques) administered by a certified yoga instructor in a group setting, given own yoga mat and book to encourage home-based practice, social gathering and tea offered to encourage group discussion		

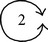	15-minute orientation and education session delivered by a certified yoga instructor, includes identification and list of community-based and online options to practise yoga		

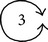	30-minute of asana training with 15-minute orientation of free and unlimited access to online yoga classes (http://www.myyogaonline.com)		

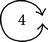	120-minute yoga workshop administered by a certified yoga instructor in a group setting, orientation and education session to include pranayama, dhyana, asana (physical postures), social gathering and tea offered to encourage group discussion		

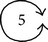	3 × 60-minute yoga classes (per week) including pranayama, asana and dhyana administered by a certified yoga instructor in a group setting, social gathering and tea offered to encourage group discussion		

**Table 2:** Yoga Intervention Descriptions.

Session number	Section title	Duration	Key components	Lesson plan outline (i.e., how many minutes of what component?)

*Group A*: *Low-dose yoga intervention *				

1	Introduction to yoga	15 min	What is yoga? How and why practice yoga? Discuss resources and how to access community-based or affordable yoga	What is Yoga? Connection of mind and body, with others and your environment; awareness. Why? To create ease, balance, and awareness; calm the mind; tool to manage symptoms and feelings; stress management. No one way to practice yoga; many styles and variations. The right way is your way. Listen to your body, always, over and above any teacher. When taking class feel free to always rest when you need it or modify postures. You are the only one who knows how your body feels. If it does not feel right, it is not right. You can always talk to the teacher before class and let them know how you are feeling. Give them as little/much info as you want. This will help them to help you. Resource list to access other yoga

1	Yoga training	30 min	Pranayama techniques; Satsang	4 minutes: introduction and overview of class direction, check-in with students (either individually or collectively) 4 minutes: breath witness/awareness 12 minutes: pranayama 10 minutes: Satsang, tea, and group discussion Note: pranayama to be done in Savasana or supported restorative, to be picked by teacher. Teacher can pick pranayama based on the feel of the room and energy level of students. Please pick from the following list. To use belly breath, sectional breathing, sighing out breathing, brahmari, and sitkari Not recommended: breath holding (causes dizziness) and nadi shodhana

*Group B*: *Medium-dose yoga intervention *				

1	Introduction to yoga and online membership	15 min	What is yoga? How and why practice yoga? Introduce membership to http://www.myyogaonline.com and give pamphlet for community-based resources	Similar to Group A, with addition of online yoga orientation Talk about “myyogaonline” website; instruct to search on the left side of screen for Hatha or restorative yoga classes or specific classes for anxiety, breathing, detoxification, gentle, relaxation, or therapeutics. Emphasise that the student only does what they feel comfortable with. One of the offerings of yoga is learning to listen to your own body and then having the respect to listen to it. If it feels good, great. If it feels bad, adjust/modify or stop.

1	Yoga training	30 min	Pranayama techniques	Same as low-dose (see above)

1	Yoga training	30 min	Asana practice and safety training	All very gentle asana 10 minutes: restorative yoga asana, floor-based 15 minutes: active yoga asana, upright and on feet (muscle retention and very easy balancing) 10 minutes: restorative yoga asana, floor-based 10 minutes: guided meditation, dhyana

2	Yoga workshop	120 min	Pranayama, asana, and dhyana techniques	10 minutes: welcome, check-in 10 minutes: witness/awareness and pranayama 20 minutes: restorative yoga asana, floor-based 15 minutes: active yoga asana, upright and on feet 20 minutes: restorative yoga asana, floor-based 10 minutes: guided meditation, dhyana 10 minutes: Satsang, tea, and group discussion 25 minutes to be used at teachers discretion based on class

*Group C: High-dose yoga intervention *				

1	Introduction to yoga and online membership	15 min	What is yoga? How and why practice yoga? Introduce membership to http://www.myyogaonline.com and give pamphlet for community-based resources	Same as medium-dose (see above)

1	Yoga training	30 min	Pranayama techniques; Satsang	Same as low- and medium-dose (see above)

1	Yoga training	30 min	Asana practice and safety training	Same as medium-dose (see above)

2 to 12	Yoga training	60 min	Pranayama, asana, and dhyana techniques; Satsang	3 minutes: welcome, check-in 2 minutes: seated pranayama exercise 10 minutes: restorative yoga asana, floor-based 15 minutes: active yoga asana, upright, and on feet 10 minutes: restorative yoga asana, floor-based 10 minutes: guided meditation, dhyana 10 minutes: Satsang, tea, and group discussion

**Table 3:** Participant characteristics.

Participant	Age at consent	Gender		Tumour location	Stage of cancer					Conventional treatment status				
		Male	Female		I	II	III	IV	n.r	Chemotherapy	Radiotherapy	Hormone therapy	Active surveillance	After surgery
A1	66	1		Tongue	1					1	1			
A2	60		1	Breast		1					1	1		
A3	51		1	Breast		1				1	1	1		
A4	40		1	Breast			1			1				
A5	41		1	Colorectal-lung	1								1	

Group A total		1	4		2	2	1	0	0	3	3	2	1	0
Average	51.6													

B1	72	1		Prostate			1				1		1	
B2	61		1	Abdomen				1				1	1	1
B3	43		1	Breast	1					1		1		
B4	51		1	Colorectal			1			1	1			
B5	59		1	Skin (forearm)					1		1			

Group B total		1	4		1	0	2	1	1	2	3	2	2	1
Average	57.2													

C1	57	1		Prostate	1								1	
C2	52		1	Breast			1			1	1		1	
C3	33		1	Brain			1			1	1			
C4	45		1	Breast		1						1		1
C5	38	1		Blood					1					

Group C total		2	3		1	1	2	0	1	2	2	1	2	1
Average	45													

Overall	**4**	**11**	**4**	**3**	**5**	**1**	**2**	**7**	**8**	**5**	**5**	**2**
Average	**51.3**											

n.r: not reported.

**Table 4:** Result of participant-reported attendance, satisfaction, and yoga utility.

Participant	Number of total yoga sessions	Number of online yoga sessions practised	Amount of satisfaction participating in yoga (0 = none, 10 = very)	Amount of satisfaction doing yoga with teacher (0 = none, 10 = very)	Amount of satisfaction using online yoga	Felt not being offered enough yoga	Would prefer more online yoga	Right amount of yoga	Too much yoga	Utility of yoga (0 = none, 10 = very)	Disutility of yoga (0 = none, 10 = very)
A1	1	0	8	7	—					8	5
A3	6	4	8	8	8	1				8	0
A4	1	0	1	1	5	1				8	0
A5	8	6	10	10	9	1				10	2

Average	**4**	**2.5**	**6.8**	**6.5**	**7.3**					**8.5**	**1.8**
Group total						**3**	**0**	**0**	**0**		

B1	4	0	8	9	0	1				8	0
B2	2	0	7	9	5	1				7	2
B3	10	0	5	10	0	1				8	4
B5	4	0	8	8	—	1				6	1

Average	**5**	**0**	**7**	**9**	**1.7**					**7.3**	**1.8**
Group total						**4**	**0**	**0**	**0**		

C1	10	0	9	9	—				1	8	0
C2	24	0	10	10	—	1				10	7
C3	5	0	6	7	7			1		8	2
C4	12	0	9	9	0	1				10	0

Average	**12.8**	**0**	**8.5**	**8.8**	**3.5**					**9**	**2.3**
Group total						**2**		**1**	**1**		

—: not reported.

**Table 5:** Summary of QOL-CA/CS results.

Participants	QOL-CA/CS	QOL-CA/CS	Change in Results		Mean Difference	Approaching Clinical Significance^*∗*^	Incomplete Data
	Baseline	Post-Intervention	Improve	Decline			
A1	6.1	7.0	•		1.0	•	
A2	5.8	—			—		•
A3	4.2	4.4	•		0.2		
A4	3.5	2.8		•	−0.8	•	
A5	5.8	8.0	•		2.2	•	

Group A Average (SD)	4.9 (1.2)	5.5 (2.4)			0.7 (1.3)		
95% CI	2.9 to 6.8	1.7 to 9.4			−1.4 to 2.7		

B1	7.9	7.3		•	−0.7		
B2	5.9	7.0	•		1.1	•	
B3	5.1	4.7		•	−0.4		
B4	4.2	—			—		•
B5	5.6	5.3		•	−0.3		

Group B Average (SD)	6.1 (1.2)	6.1 (1.2)			−0.1 (0.8)		
95% CI	4.2 to 8.1	4.1 to 8.0			−1.4 to 1.1		

C1	6.8	6.6		•	−0.2		
C2	3.8	4.6	•		0.9	•	
C3	6.9	7.1	•		0.3		
C4	4.0	3.8		•	−0.2		
C5	4.1	—					•

Group C Average (SD)	5.4 (1.7)	5.5 (1.6)			0.2 (0.5)		
95% CI	2.7 to 8.1	3.0 to 8.6			−0.7 to 1.0		

Overall Average (SD)	5.5 (1.4)	5.7 (1.7)			035 (0.9)		
95% CI	4.6 to 6.3	4.7 to 6.8			−0.3 to 0.8		
Total			6	6			3

^*∗*^The minimum clinical difference (MCID) score was not provided by instrument authors; based on a literature of similar scales of HRQoL, approaching clinical significance is defined here as mean difference >0.8; SD = standard deviation; CI = confidence interval; overall and group analysis of complete data sets only (*n* = 12; *n* = 4 per group).

**Table 6:** Summary of QOL-CA/CS subscale results.

Physical Wellbeing Subscale				Psychological Wellbeing Subscale			
Participants	Baseline	Post-intervention	Mean Difference	Participants	Baseline	Post-intervention	Mean Difference
Group A				Group A			
Average (SD)	5.82 (1.27)	5.19 (3.05)	−0.63 (2.66)	Average (SD)	4.85 (1.71)	5.73 (2.18)	0.88 (1.09)
95% CI	3.80 to 7.84	0.34 to 9.77	−3.60 to 4.86	95% CI	2.13 to 7.57	2.24 to 9.19	−2.61 to 0.85
Group B				Group B			
Average (SD)	7.21 (2.25)	7.88 (1.76)	0.67 (0.78)	Average (SD)	5.97 (1.20)	5.51 (1.08)	−0.47 (0.96)
95% CI	3.64 to 10.78	5.08 to 10.68	−0.57 to 1.91	95% CI	4.07 to 7.87	3.79 to 7.23	−2.00 to 1.06
Group C				Group C			
Average (SD)	7.32 (2.59)	6.60 (2.81)	−0.72 (1.54)	Average (SD)	4.44 (1.74)	4.89 (1.29)	0.45 (0.45)
95% CI	3.20 to 11.44	2.13 to 11.07	−3.18 to 1.74	95% CI	1.67 to 7.21	−1.56 to 2.54	−0.27 to 1.17

Overall				Overall			
Average (SD)	6.78 (2.04)	6.55 (2.59)	−0.23 (1.76)	Average (SD)	5.09 (1.57)	5.37 (1.48)	0.29 (0.99)
95% CI	5.49 to 8.07	4.90 to 8.20	−1.35 to 0.89	95% CI	4.09 to 6.09	4.43 to 6.31	−0.34 to 0.92

Social Wellbeing Subscale				Spiritual Wellbeing Subscale			
Participants	Baseline	Post-intervention	Mean Difference	Participants	Baseline	Post-intervention	Mean Difference

Group A				Group A			
Average (SD)	4.94 (1.34)	5.29 (2.74)	0.35 (1.63)	Average (SD)	3.82 (1.18)	5.78 (1.97)	1.96 (1.15)
95% CI	2.80 to 7.08	0.93 to 9.65	−2.24 to 2.94	95% CI	1.94 to 5.70	2.65 to 8.91	0.13 to 3.79
Group B				Group B			
Average (SD)	6.29 (1.85)	6.47 (1.92)	0.19 (1.02)	Average (SD)	5.14 (1.35)	5.07 (1.56)	−.07 (1.74)
95% CI	3.35 to 9.23	3.42 to 9.52	−1.43 to 1.81	95% CI	3.00 to 7.28	2.59 to 7.55	−2.70 to 2.84
Group C				Group C			
Average (SD)	5.07 (2.06)	4.86 (1.86)	−0.21 (0.78)	Average (SD)	5.86 (1.62)	6.68 (1.78)	0.83 (0.55)
95% CI	1.79 to 8.35	1.90 to 7.82	−1.45 to 1.03	95% CI	3.28 to 8.44	3.85 to 9.51	−0.05 to 1.66

Overall				Overall			
Average (SD)	5.43 (1.73)	5.54 (2.13)	0.11 (1.11)	Average (SD)	5.18 (1.68)	5.85 (1.75)	0.67 (1.63)
95% CI	4.43 to 6.53	4.19 to 6.89	−0.59 to 0.81	95% CI	4.11 to 6.25	4.74 to 6.96	−0.42 to 1.70

SD = standard deviation; CI = confidence interval; complete data sets only (*n* = 12); subgroups (*n* = 4).
